# Does low dose of etoricoxib play pre-emptive analgesic effect in third molar surgery? A randomized clinical trial

**DOI:** 10.1186/s12903-021-01837-0

**Published:** 2021-09-23

**Authors:** Long Xie, Lei Sang, Zhi Li

**Affiliations:** 1 The State Key Laboratory Breeding Base of Basic Science of Stomatology (Hubei-MOST) and Key Laboratory of Oral Biomedicine Ministry of Education, School and Hospital of Stomatology, Wuhan University, Wuhan, China; 2 Department of Oral and Maxillofacial Surgery, School and Hospital of Stomatology, Wuhan University, 237 Luoyu Road, Wuhan, 430079 China; 3 Department of Stomatology, Suzhou Vocational Health College, Suzhou, China

**Keywords:** Pre-emptive analgesia, Etoricoxib, Third molar, Extraction, Post-operative pain

## Abstract

**Background:**

How to prevent pain after the extraction of impacted teeth is a serious challenge for all patients. The purpose of this clinical trial was to investigate whether pre-emptive low dose of etoricoxib can reduce postoperative pain in patients undergoing third molars surgery.

**Methods:**

Patients were randomised to receive etoricoxib 60 mg or placebo 30 min before surgery. Post-operative pain was recorded using a visual analogue scale during 24 h within the post-operative period. The total dose of ibuprofen rescue intake was recorded. Kaplan–Meier curves and log-rank analyses were used to evaluate the proportion of patients without rescue analgesic.

**Results:**

Scores for the post-operative pain in the etoricoxib group were significantly lower than those in the placebo group during first 12 h (*p* < 0.05). The number of patients without analgesic rescue medication was significantly lower in the etoricoxib group than in the placebo group. The average amount of rescue medication in the etoricoxib group (0.4 ± 0.9 dose) was lower than that in the placebo group (1.1 ± 0.9 doses, *p* = 0.004). Etoricoxib resulted in the long-term survival of patients without rescue analgesic (*p* < 0.001).

**Conclusions:**

This study revealed that etoricoxib has a substantial pre-emptive analgesic effect, resulting in the reduced use of analgesics after third molar removal.

*Trial registration*: Registered on ChiCTR1900024503. Date of Registration: 13/07/2019.

**Supplementary Information:**

The online version contains supplementary material available at 10.1186/s12903-021-01837-0.

## Background

Acute pain caused by mandibular third molar removal is widely used to evaluate the efficacy of analgesics. Treatment of moderate or severe pain is usually given after the operation to reduce the dispersion of pain intensity measures by only including patients who need analgesics [1]. Agents, such as nonsteroidal anti-inflammatory drugs (NSAIDs), acetaminophen and opioids, are effective in treating acute pain [2].

NSAIDs contain two cyclooxygenase isoforms, namely, cyclooxygenase-1 (COX-1) and cyclooxygenase-2 (COX-2) [3]. COX-1 is constitutively expressed in tissues and promotes the synthesis of prostaglandin (PG). The gastric and renal side effects of NSAIDs may be due to their indirect influence on PGE2, which has a cytoprotective effect in the gastrointestinal system, as well as on PGE2 and PGI2, which regulate renal blood flow [4]. COX-2 is also located in certain healthy tissues, but this isoform is especially induced by inflammatory stimulus or mitogen in some tissues. The expression of COX-2 may be related to the synthesis of PG, which induces responses to pathological processes, such as pain, fever and inflammation [5]. Although nonselective NSAIDs are first-line analgesics, their additional inhibition of COX-1 increases the hazard of gastrointestinal toxicity and thus limits their administration [6].

Etoricoxib, as the COX-2 selective class of NSAIDs, can provide patients with effective painkillers who do not benefit adequately from first-line therapies [7]. As the second generation of the selective class of NSAIDs, in various cells and whole blood tests, etoricoxib is more than 100 times selective for COX-2 than for COX-1 and is obviously less active against COX-1 than other selective COX-2 inhibitors [8]. This drug is also effective in relieving pain during dental procedures, total abdominal hysterectomy, periodontal surgery and therapeutic knee arthroscopy [9–12]. This type of NSAID is a safe and effective drug that controls postoperative pain with minimal side effects.

The pre-emptive analgesic of large doses of etoricoxib (120 mg) has been demonstrated to reduce pain after tooth extraction surgery [13]. At present, few studies were conducted on the use of low-dose etoricoxib (60 mg) after the operation of impacted teeth, and the results cannot provide a basis for clinical practice [1]. Our clinical trial was performed to evaluate the efficacy of a low dose of etoricoxib (60 mg) on alleviating pain after third molar surgery.

## Methods

### Study design and sample

This study was designed as a randomized, parallel, double-blinded and placebo-controlled clinical trial from August 2019 to July 2020. Healthy patients scheduled to undergo surgical removal of an impacted horizontal mandibular third molar (Winter classification) were eligible for participation. Patients were included if they were older than 18 years old, had horizontal impacted teeth, did not take analgesics or anti-inflammatory drugs a week prior to the study. Patients were excluded if they took NSAIDs and COX-2 inhibitors; were pregnant or nursing; had other serious diseases, such as liver, kidney and cardiovascular diseases; had ulcers or bleeding in the digestive tract; had the history of GI bleeding and gastritis; had inability to express subjective discomfort symptoms; and suffered from dental caries or apical periodontitis with the adjacent teeth. The Patients were given standardised participant information sheets and signed written informed consent for their participation.

All patients were informed about the study protocol and possible risks prior to any procedure. The patients were randomly divided into etoricoxib and placebo groups by Excel software. The etoricoxib group orally receive etoricoxib tablet 60 mg (Merck&Co., Inc) or placebo group (tablet, without active drug) 30 min before surgery. The operator and the patients were blinded to the type of drugs administered. To prevent postoperative infection, 0.5 g dose of amoxicillin and 0.4 g dose of metronidazole tablets were taken orally, 3 times a day, for 5 days, and 1.0 g dose of azithromycin was given once a day for 5 days if allergic to amoxicillin.

### Randomisation and blinding

The patients were divided into two groups (placebo and 60 mg of etoricoxib) by Excel software to achieve randomisation. Allocation concealment was maintained using a sealed opaque envelope. The research assistant prepared the study drugs for the clinic nurses according to the randomisation list. A dedicated nurse, as non-treatment group members, gave the study drugs sealed in a similar package to the patients 30 min before the surgery.

### Sample size calculation

Based on the mean pain scores of previous study [14] (etoricoxib 1.9 ± 1.5 and placebo 3.6 ± 1.9), a minimum sample size of twenty-eight patients per group were required to conduct this clinical trial and statistically reject the null hypothesis with 95% power. For this sample calculation, the type 1 error associated with the test was 0.05; χ2 test without correction was used to evaluate the null hypothesis. The sample unit used in the present study was the third molar.

### Interventions

All operations were performed by the same attending doctor to minimise differences between operators. The same local anaesthesia technique was performed on the patients. The anaesthesia of inferior alveolar, lingual and buccal nerves block was performed with 2% lidocaine. For local infiltration anaesthesia, 4% atecaine and 1:100,000 epinephrine (Septanest, Septodont, France) were used to reduce intraoperative bleeding. Both groups received the same surgical procedure to reduce surgery-related bias. The buccal mucoperiosteal flap was elevated, the bone was removed and the tooth was sectioned. After the tooth was extracted, the alveolar tissue was scraped and rinsed with sterile saline solution. The wound was sutured with a 4–0 silk, and the suture was removed 1 week after the surgery. The operative time was calculated from flap dissection and suture after tooth extraction.

### Pain assessment

An 11-point (0 to 10) visual analogue score (VAS) was used to evaluate pain (0, no pain; 1–3, mild pain; 4–6, moderate pain; 7–9, severe pain; and 10, miserable pain.

). Postoperative pain was assessed and scored at 2, 4, 6, 8, 12 and 24 h after the operation. Ibuprofen (300 mg) was prescribed as the emergency analgesic only in the case of VAS > 3. The patients were asked to record their total consumption of ibuprofen (amount of tablets) within the first 24 h (at 2, 4, 6, 8, 12 and 24 h) and the time of the first analgesic rescue medication after the procedure was completed.

### Statistical analysis

Data were analysed with SPSS software (SPSS, Inc., USA). Independent t-test and Chi-square were used to determine significant difference between the two groups. The parametric outcomes were expressed as mean ± standard deviation (SD). The survival curves were estimated by the Kaplan–Meier method, and log-rank test was applied to compare differences between curves. *p* value less than 0.05 was considered statistically significant.

## Results

### Sample characterisation

**Fig. 1:**
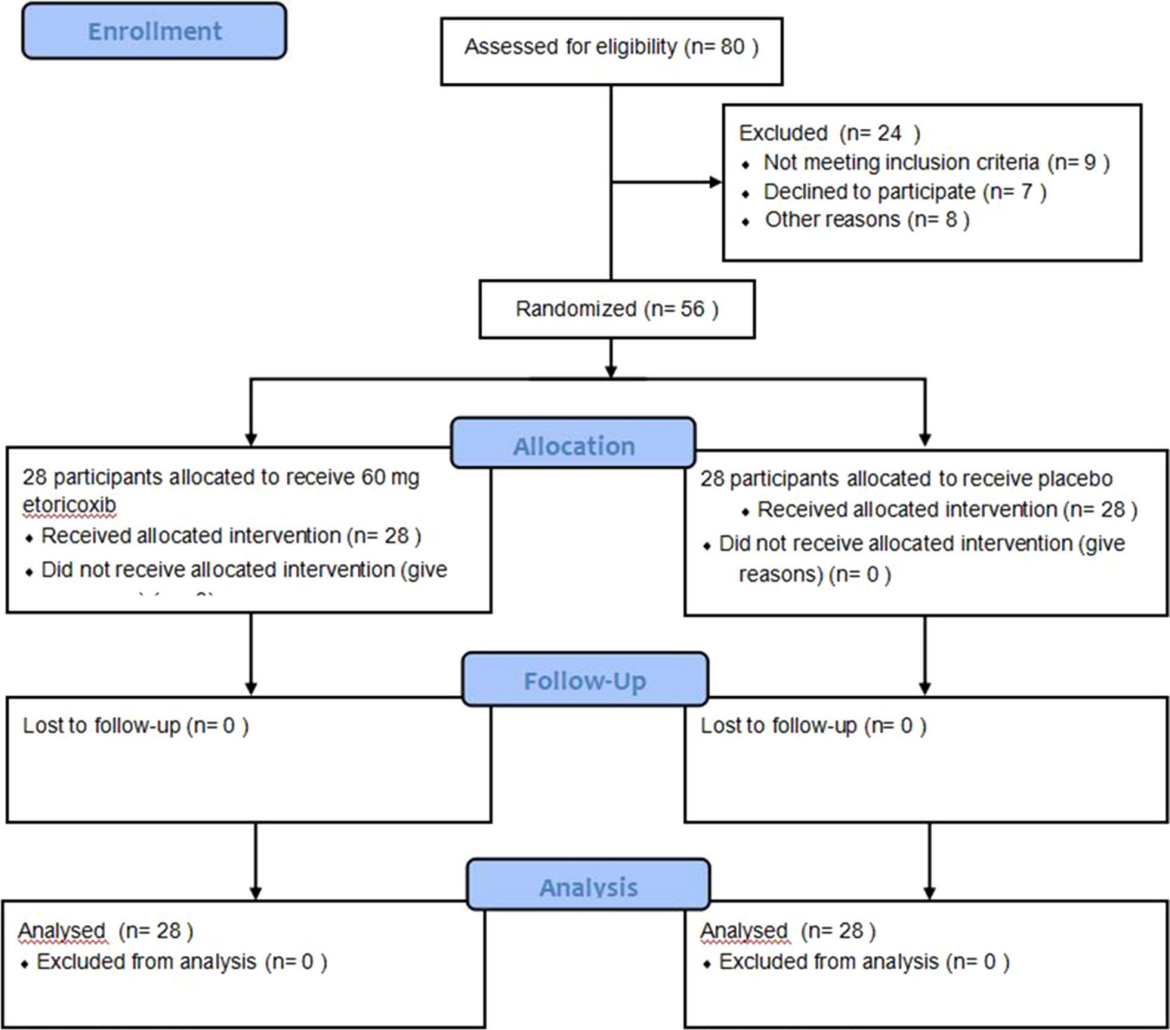
Flowchart of participants recruitment according to the CONSORT protocol

Eighty patients were considered eligible (Fig. 1). Twenty-four individuals were excluded because they did not meet the study criteria. Finally, 56 patients (male and female aged between 18 and 45 years) were selected for the clinical trial and randomly divided into etoricoxib and control groups. None of the drugs used had reported side effects. No differences were detected in terms of age, gender, BMI and duration of the operation between the two groups (Table 1). Table 2 shows the level of impaction (Pell–Gregory classification) for the patients in each of the study groups. There was no significant difference between the two groups for any parameter.

**Table 1:** Demographic data

	Placebo	Etoricoxib	*p* value
Age(years)	28.1 ± 5.0	29.4 ± 5.0	.368^a^
BMI(kg/m^2^)	21.4 ± 3.0	22.6 ± 3.2	.174^a^
Gender (M/F)	10/18	13/15	.587^b^
Duration of the operation (minutes)	14.0 ± 4.8	15.8 ± 5.3	.191^a^

Data are presented as mean (standard deviation) or number

BMI, body mass index

^a^By t test

^b^By χ^2^ test

**Table 2:** Pell and Gregory classification

	2B	2C	χ^2^	*p* value
Placebo (n = 29)	18	11	0.284	0.790
Etoricoxib (n = 29)	16	13		

### Pain (VAS) analysis

**Fig. 2:**
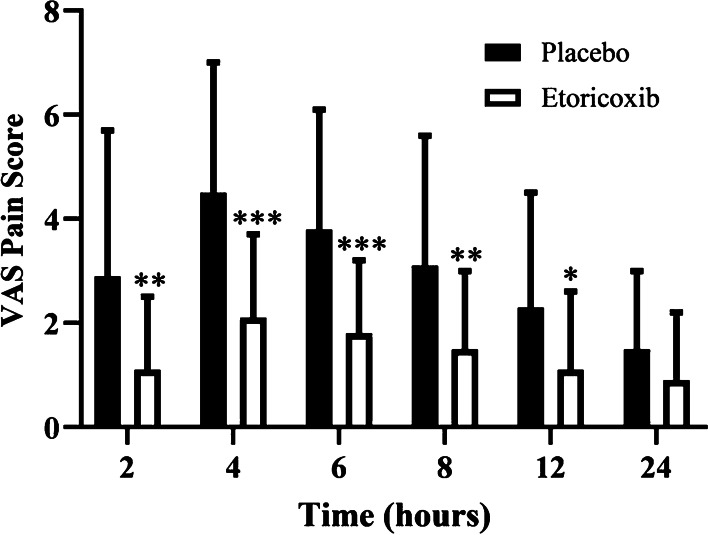
Pain scores at 2, 4, 6, 8, 12 and 24 h after surgery as measured on visual analog scale (VAS)

Table 3 and Fig. 2 show the mean VAS pain score at each postoperative time. The mean VAS score in the etoricoxib group was significantly lower than that in the placebo group in the first 12 h during the follow-up period after the surgery.

**Table 3:** Average pain measurements in study groups

Timing of VAS Score, h	Placebo	Etoricoxib	*P* value
2	2.9 ± 2.8	1.1 ± 1.4	.004**
4	4.5 ± 2.5	2.1 ± 1.6	*p* < .001***
6	3.8 ± 2.3	1.8 ± 1.4	*p* < .001***
8	3.1 ± 2.5	1.5 ± 1.5	.006**
12	2.3 ± 2.2	1.1 ± 1.5	.027*
24	1.5 ± 1.5	0.9 ± 1.3	.112

Values are expressed mean ± standard deviation

VAS, visual analog pain scale

Values are expressed mean ± standard deviation or number

^*^p < 0.05 t-test between groups

^**^p < 0.01 t-test between groups

^***^p < 0.001 t-test between groups

### Rescue analgesic intake dose

Table 4 shows the average consumption of rescue analgesic during the 24 h period. Patients in the etoricoxib group required less rescue analgesic compared with those in the placebo group.

**Table 4:** Comparison of the average dosage and number of patients during 24-h rescue analgesic intake among groups

Variable	Placebo (n = 28)	Etoricoxib (n = 28)	*p* value
Number of patients who consumed the first rescue analgesic medication during the period of evaluation (24 h)	21 (75%)	8(28.6%)	.001^b^
Number (%) of patients requiring no rescue analgesic medication during the period of evaluation (24 h)	7(25%)	20(71.4%)	.001^b^
Total analgesic consumption for postoperative 24 h (tablets) (mean ± SD)	1.1 ± 0.9	0.4 ± 0.9	.004^a^

Data are presented as mean (standard deviation) or number

^a^By t test

^b^By χ^2^ test

### Interval to the first intake of ibuprofen

**Fig. 3:**
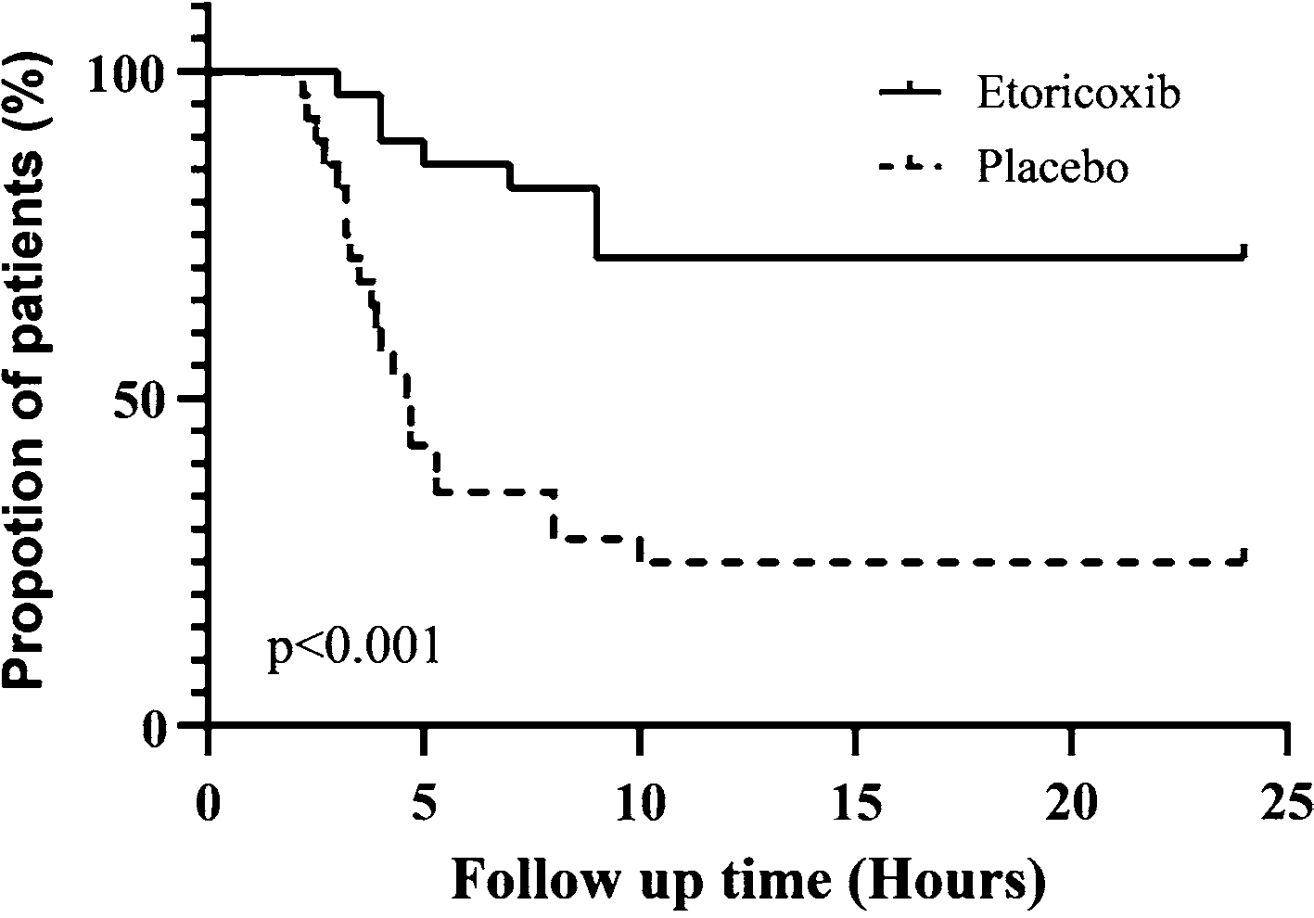
Kaplan–Meier curve for overall survival stratified by etoricoxib group and placebo group use (Log rank test)

The Kaplan–Meier curve in Fig. 3 shows the data of patients who took emergency analgesics within 24 h after the surgery. In the etoricoxib group, 71.4% of the patients did not take any painkillers after the surgery, which was 46.4% more than that in the placebo group (*P* = 0.001). The overall survival time (the interval to the first intake of ibuprofen) in the etoricoxib group was longer than that in the placebo group (*P* < 0.001).

## Discussion

This study revealed that pre-emptive oral administration of etoricoxib (60 mg) is an effective pain management strategy compared to placebo after third molar surgery. Etoricoxib (60 mg) significantly reduced the pain scores during the first 12 postoperative hours, resulting in longer overall survival time (the interval to the first intake of ibuprofen) and reduced need of rescued analgesic consumption within 24 h after the surgery.

About 60% of the patients suffered moderate pain, and 40% had severe pain, which had higher requirements for analgesia [15]. Inadequate postoperative pain management resulted in a significant deterioration in the quality of life of the patients after third molar removal [16]. Therefore, pain management after third molar removal has received extensive attention from surgeons and patients. In 2010, the Italian Society of Odontostomatological Surgery made the recommendation that pre-emptive analgesia is expected to be the most effective method for controlling postoperative pain [17].

Pre-emptive analgesia is a treatment initiated before the operation to prevent central sensitisation due to incision injury and other inflammatory reactions [18]. Pre-emptive analgesia has been demonstrated to be safe and can decrease pain in the early postoperative period [19]. This method has achieved good analgesic effects in several kinds of surgical operations. Pre-emptive analgesia of etoricoxib can reduce acute postoperative pain in patients who underwent orthopaedics surgery, laparoscopic cholecystectomy and panphotocoagulation [20–22]. Albuquerque et al.[14]. found that pre-emptive analgesia of etoricoxib (120 mg) can lead to reduce pain, trismus and oedema after third molar surgery.

A low dose of etoricoxib (60 mg) showed significant analgesic effect and minimal side effects compared to placebo. In a previous study, 500 patients with osteoarthritis (OA) were administered with 60 mg of etoricoxib once a day for 4 weeks without prior medication washout. Disability and pain interference in daily activities were significantly improved [23]. In addition, the pain and disability scores measured by the Western Ontario and McMaster’s University OA index (WOMAC) in 19 extremely elderly men with OA (mean age, 85.9 years; age range, 79–96 years) were lower after treatment with 60 mg of etoricoxib once daily for 4 weeks; no adverse events were also reported [24]. A double-blind and placebo-controlled study conducted a two-part dose-ranging clinical trial of etoricoxib (5–90 mg/day) starting at 14-week; during which, 60 mg of etoricoxib was found to have the best analgesic effect according to the WOMAC pain scale [25]. Another clinical trial during the 46-week active-comparator controlled period, drug-related laboratory adverse experience, such as increase in alanine aminotransferase and aspartate aminotransferase, were reported for 2.0%, 0.0%, 4.1% and 11.8% in the groups administered with 30, 60 and 90 mg of etoricoxib and 150 mg of diclofenac, respectively [26]. These studies indicated that a low dose of etoricoxib (60 mg) is safer than other doses.

In our study, a low dose of etoricoxib (60 mg) was more effective than placebo in preventing pain at first 12 h post-surgery. Gupta et al. discovered that a low dose of etoricoxib (60 mg) was highly effective in controlling pain during fixed orthodontic appliance therapy [27]. In an acute pain model, postoperative analgesia of low dose of etoricoxib (60 mg) can reduce pain [1]. Our research is similar to the above-mentioned study. However, according to Costa et al., pre-emptive 120 mg etoricoxib single dose provided significant analgesia compared to placebo in the first 48 h after surgery (compared to 12 h in present study), with no side effects [13]. Although pre-emptive treatment with 120 mg etoricoxib had a longer-lasting analgesic effect compared to 60 mg etoricoxib, patients in Costa and this study experienced mild pain 24 h after surgery. To reduce mild pain, increasing the dose of etoricoxib may increase the risk of adverse reactions [26]. Furthermore, in Costa's trial, the sample size was only 18 patients. If the sample size was increased, the odds of adverse reactions among patients might be increased. In this study, a pre-emptive of low dose of etoricoxib was enough to induce good analgesia with no side effects.

It should be noted that at the most pain intensity, the mean VAS measurements of treatment and placebo groups were 2.1 and 4.5 in this study. Maximum pain in this study was moderate in the placebo group. This may be related to the experience and skill of the surgeon. It is also clearly aware that the analgesic effect of low dose of etoricoxib should be specified to the mild/moderate surgery and should not be generalized for all cases at present.

PGE2 was highly expressed in the peripheral tissue and central nervous system during and after the surgery; upregulated of PGE2 was associated with increased pain scores on the VAS scale [28]. Etoricoxib reached the cerebrospinal fluid (CSF) and the surgical site in an effective concentration and reduced the production of PGE2 at the presumed site of action [29]. This process resulted in complete blockade of PGE2 production in the surgical wound and CSF. This phenomenon can lead to pain relief and reduce demands for post-operative analgesics.

The total analgesic consumption at 24 h post-operation (tablets, mean ± SD) in the etoricoxib group (0.4 ± 0.9) was less than that (1.1 ± 0.9) in the placebo group. The statistical difference between the two groups could be attributed to the analgesia, namely, etoricoxib, applied in this study. Through clinical trials, Malmstrom et al. found that patients with moderate or severe pain after removal of two or more third molars who took 120 mg of etoricoxib had reduced demand for rescue medication [30]. Steffens et al.[11]. proposed that the pre-emptive use of 120 mg of etoricoxib can relieve the post-operative pain after open-flap debridement surgery and reduce the total amount of rescue medication needed. Lower TNF-a concentration can lead to less rescue medication as a result of pre-emptive administration of 120 mg of etoricoxib after third molar removal compared with that in the placebo group [14]. In the present research, pre-emptive analgesia of low dose of etoricoxib (60 mg) can reduce the need for emergency analgesics. This clinical trial showed longer interval between taking emergency analgesics in the etoricoxib group than in the placebo group. More patients in the control group (75.0% patients) used emergency analgesics than in the etoricoxib group (28.6% patients). Malmstrom and his colleagues discovered that during the 24 h of the study period, 52.0% of patients in the etoricoxib group (60 mg) and 81.6% in the placebo group used rescue medication [1]. Compared with Malmstrom’s study, the present study showed that less patients took emergency analgesics because of removal of one third molars and the pre-emptive administration of etoricoxib. The interval between the first use of emergency analgesics was 12.1 h (etoricoxib 60 mg group) and 2.1 h (placebo group) [1]. These data are consistent with our finding that patients in the etoricoxib group had not a degree of pain who necessitate intervention by analgesics compared with those in the placebo group. The long-term analgesic effect could be related to the elimination half-life of etoricoxib in plasma for 25–30 h [31].

## Limitation

This was a preliminary study with indispensable limitations. On the one hand, this study lacked a positive control group. This study found that low dose of etoricoxib was helpful to reduce postoperative pain after tooth extraction, but NSAID analgesics could also achieve this effect. Secondly, the postoperative follow-up time was only 24 h, and the pain assessment after 24 h was not observed. In the future study, we will extend the follow-up time and focus on the effect of etoricoxib compared with other NSAID drugs on postoperative pain after tooth extraction.

## Conclusions

This study revealed that a low dose of etoricoxib has pre-emptive analgesic effect, resulting in the reduced use of analgesics after third molar removal.

## Supplementary Information

**Additional file 1.** Trail protocol.

## Data Availability

The datasets used and/or analysed during the current study are available from the corresponding author on reasonable request.

## Abbreviations

VAS: Visual analog scale
NSAIDs: Nonsteroidal anti-inflammatory drugs
COX-1: Cyclooxygenase-1
SD: Standard deviation
OA: Osteoarthritis
WOMAC: The Western Ontario and McMaster’s University OA index
PG: Prostaglandin

## References

[1] Malmstrom K, Sapre A, Couglin H, Agrawal NG, Mazenko RS, Fricke JR (2004). Etoricoxib in acute pain associated with dental surgery: a randomized, double-blind, placebo- and active comparator-controlled dose-ranging study. Clin Ther.

[2] Mehrabi M, Allen JM, Roser SM (2007). Therapeutic agents in perioperative third molar surgical procedures. Oral Maxillofac Surg Clin North Am.

[3] Seibert K, Zhang Y, Leahy K, Hauser S, Masferrer J, Isakson P (1997). Distribution of COX-1 and COX-2 in normal and inflamed tissues. Adv Exp Med Biol.

[4] Süleyman H, Demircan B, Karagöz Y (2007). Anti-inflammatory and side effects of cyclooxygenase inhibitors. Pharmacol Rep.

[5] Meade EA, Smith WL, DeWitt DL (1993). Differential inhibition of prostaglandin endoperoxide synthase (cyclooxygenase) isozymes by aspirin and other non-steroidal anti-inflammatory drugs. J Biol Chem.

[6] Wolfe MM, Lichtenstein DR, Singh G (1999). Gastrointestinal toxicity of nonsteroidal antiinflammatory drugs. N Engl J Med.

[7] Brown JD, Daniels SE, Bandy DP, Ko AT, Gammaitoni A, Mehta A, Boice JA, Losada MC, Peloso PM (2013). Evaluation of multiday analgesia with etoricoxib in a double-blind, randomized controlled trial using the postoperative third-molar extraction dental pain model. Clin J Pain.

[8] Riendeau D, Percival MD, Brideau C, Charleson S, Dubé D, Ethier D, Falgueyret JP, Friesen RW, Gordon R, Greig G, Guay J, Mancini J, Ouellet M, Wong E, Xu L, Boyce S, Visco D, Girard Y, Prasit P, Zamboni R, Rodger IW, Gresser M, Ford-Hutchinson AW, Young RN, Chan CC (2001). Etoricoxib (MK-0663): preclinical profile and comparison with otheragents that selectively inhibit cyclooxygenase-2. J Pharmacol Exp Ther.

[9] Daniels SE, Bandy DP, Christensen SE, Boice J, Losada MC, Liu H, Mehta A, Peloso PM (2011). Evaluation of the dose range of etoricoxib in an acute pain setting using the postoperative dental pain model. Clin J Pain.

[10] Viscusi ER, Frenkl TL, Hartrick CT, Rawal N, Kehlet H, Papanicolaou D, Gammaitoni A, Ko AT, Morgan LM, Mehta A, Curtis SP, Peloso PM (2011). Perioperative use of etoricoxib reduces pain and opioid side-effects aftertotal abdominal hysterectomy: a double-blind, randomized, placebo-controlled phase III study. Curr Med Res Opin.

[11] Steffens JP, Santos FA, Sartori R, Pilatti GL (2010). Preemptive dexamethasone and etoricoxib for pain and discomfort prevention after periodontal surgery: a double-masked, crossover, controlled clinical trial. J Periodontol.

[12] Lierz P, Losch H, Felleiter P (2012). Evaluation of a single preoperative dose of etoricoxib for postoperativepain relief in therapeutic knee arthroscopy: a randomized trial. Acta Orthop.

[13] Costa FW, Soares EC, Esses DF, Silva PG, Bezerra TP, Scarparo HC, Ribeiro TR, Fonteles CS (2015). A split-mouth, randomized, triple-blind, placebo-controlled study to analyze the pre-emptive effect of etoricoxib 120 mg on inflammatory events following removal of unerupted mandibular third molars. Int J Oral Maxillofac Surg.

[14] Albuquerque AFM, Fonteles CSR, do Val DR, Chaves HV, Bezerra MM, Pereira KMA, de Barros Silva PG, de Lima BB, Soares ECS, Ribeiro TR, Costa FWG (2017). Effect of pre-emptive analgesia on clinical parameters and tissue levels of TNF-α and IL-1β in third molar surgery: a triple-blind, randomized, placebo-controlled study. Int J Oral Maxillofac Surg.

[15] Averbuch M, Katzper M (2003). Severity of baseline pain and degree of analgesia in the third molar post-extraction dental pain model. Anesth Analg.

[16] Duarte-Rodrigues L, Miranda EFP, Souza TO, de Paiva HN, Falci SGM, Galvão EL (2018). Third molar removal and its impact on quality of life: systematic review and meta-analysis. Qual Life Res.

[17] Annibali S, De Biase A, Pippi R, Sfasciotti GL, Italian Society of Odontostomatological Surgery (2011). A consensus conference on management of the lower third molar Italian Society of Odontostomatological Surgery. Minerva Stomatol.

[18] Kissin I (2000). Preemptive analgesia. Anesthesiology.

[19] Van Backer JT, Jordan MR, Leahy DT, Moore JS, Callas P, Dominick T, Cataldo PA (2018). Preemptive analgesia decreases pain following anorectal surgery: a prospective, randomized, double-blinded. Placebo-controlled trial. Dis Colon Rectum.

[20] Boonriong T, Tangtrakulwanich B, Glabglay P, Nimmaanrat S (2010). Comparing etoricoxib and celecoxib for preemptive analgesia for acute postoperative pain in patients undergoing arthroscopic anterior cruciate ligament reconstruction: a randomized controlled trial. BMC Musculoskelet Disord.

[21] Ko-Iam W, Paiboonworachat S, Pongchairerks P, Junrungsee S, Sandhu T (2016). Combination of etoricoxib and low-pressure pneumoperitoneum versus standard treatment for the management of pain after laparoscopic cholecystectomy: a randomized controlled trial. Surg Endosc.

[22] Nascimento VP, Primiano HP, Maia OO, Pelayes D, Takahashi WY (2012). Analgesic effect of etoricoxib (Arcoxia®) 120 mg during retinal laser photocoagulation. Eur J Ophthalmol.

[23] Lin HY, Cheng TT, Wang JH, Lee CS, Chen MH, Lei V, Lac C, Gammaitoni AR, Smugar SS, Chen WJ (2010). Etoricoxib improves pain, function and quality of life: results of a real-world effectiveness trial. Int J Rheum Dis.

[24] Huang WN, Tso TK (2018). Etoricoxib improves osteoarthritis pain relief, joint function, and quality of life in the extreme elderly. Bosn J Basic Med Sci.

[25] Gottesdiener K, Schnitzer T, Fisher C, Bockow B, Markenson J, Ko A, DeTora L, Curtis S, Geissler L, Gertz BJ (2002). Results of a randomized, dose-ranging trial of etoricoxib in patients with osteoarthritis. Rheumatology (Oxford).

[26] Curtis SP, Bockow B, Fisher C, Olaleye J, Compton A, Ko AT, Reicin AS (2005). Etoricoxib in the treatment of osteoarthritis over 52-weeks: a double-blind, active-comparator controlled trial NCT00242489. BMC Musculoskelet Disord.

[27] Gupta M, Kandula S, Laxmikanth SM, Vyavahare SS, Reddy SB, Ramachandra CS (2014). Controlling pain during orthodontic fixed appliance therapy with non-steroidal anti-inflammatory drugs (NSAID): a randomized, double-blinded, placebo-controlled study. J Orofac Orthop.

[28] Buvanendran A, Kroin JS, Berger RA, Hallab NJ, Saha C, Negrescu C, Moric M, Caicedo MS, Tuman KJ (2006). Upregulation of prostaglandin E2 and interleukins in the central nervous system and peripheral tissue during and after surgery in humans. Anesthesiology.

[29] Renner B, Zacher J, Buvanendran A, Walter G, Strauss J, Brune K (2010). Absorption and distribution of etoricoxib in plasma, CSF, and wound tissue in patients following hip surgery—a pilot study. Naunyn Schmiedebergs Arch Pharmacol.

[30] Malmstrom K, Ang J, Fricke JR, Shingo S, Reicin A (2005). The analgesic effect of etoricoxib relative to that of cetaminophen analgesics: a randomized, controlled single-dose study in acute dental impaction pain. Curr Med Res Opin.

[31] Agrawal NG, Porras AG, Matthews CZ, Rose MJ, Woolf EJ, Musser BJ, Dynder AL, Mazina KE, Lasseter KC, Hunt TL, Schwartz JI, McCrea JB, Gottesdiener KM (2003). Single- and multiple-dose pharmacokinetics of etoricoxib, a selective inhibitor of cyclooxygenase-2, in man. J Clin Pharmacol.

