# Supplementary material for: Does low dose of etoricoxib play pre-emptive analgesic effect in third molar surgery? A randomized clinical trial
Source: BMC Oral Health. 2021 Sep 23;21:462. doi: 10.1186/s12903-021-01837-0 (PMC8459478; doi:10.1186/s12903-021-01837-0)
Supplement: Supplementary file 1 — Additional file 1. Trail protocol. [file 12903_2021_1837_MOESM1_ESM.docx]

**Does** **low dose of etoricoxib play pre-emptive analgesic effect in third molar surgery? A** **randomized clinical trial**

Long Xie DDS, MD ^1,2&^, Lei Sang MD ^3&^, Zhi Li DDS, MD, PhD^1,2^

^1^ The State Key Laboratory Breeding Base of Basic Science of Stomatology (Hubei-MOST) & Key Laboratory of Oral Biomedicine Ministry of Education, School & Hospital of Stomatology, Wuhan University, Wuhan, China;

^2^ Department of Oral and Maxillofacial Surgery, School and Hospital of Stomatology, Wuhan University, Wuhan, China;

^3^ Department of Stomatology, Suzhou Vocational Health College, Suzhou, China.

^&^Long Xie and Lei Sang contributed equally to this work and are considered as joint first authors.

Zhi Li is considered as corresponding authors. Address correspondence and reprint requests to Dr. Li: Department of Oral and Maxillofacial Surgery, School and Hospital of Stomatology, Wuhan University, 237 Luoyu Road, Wuhan 430079, China; E-mail: zhili@whu.edu.cn. Tel: 86-27-87686216; Fax: 86-27-87873260.

Summary

Pre-emptive approaches focus on preventing post-operative algesic flare and moderating or blocking the occurrence of hyperalgesic states. Pre-emptive analgesic strategies aim to control or prevent central sensitization.

Acute postoperative toothache is painful and uncomfortable, and it is considered to be the most ideal model for studying analgesia. Impacted tooth extraction surgery is widely used to study the analgesic effects of single or combined drugs. Oral painkillers are taken after routine compression to stop bleeding for 1 hour after tooth extraction. At this time, oral painkillers often take a while to take effect. During this time, the patient has already felt obvious pain. After oral analgesics before tooth extraction, the analgesics can achieve preemptive analgesia after the analgesic drugs take effect, which helps to reduce postoperative pain. investigate whether preemptive low dose of etoricoxib (60mg) can reduce postoperative pain in patients undergoing third molars surgery. Patients were randomised to receive etoricoxib 60 mg or placebo 30 minutes before surgery. Post-operative pain was recorded using a visual analogue scale during 24 h within the post-operative period. The total dose of ibuprofen rescue intake was recorded. Kaplan-Meier curves and log-rank analyses were used to evaluate the proportion of patients without rescue analgesic. Based on these data, the effect of prophylactic low-dose etocoxib on postoperative pain after extraction of impacted teeth can be evaluated.

Introduction

Acute pain caused by mandibular third molar removal is widely used to evaluate the efficacy of analgesics. Treatment of moderate or severe pain is usually given after the operation to reduce the dispersion of pain intensity measures by only including patients who need analgesics [1]. Agents, such as nonsteroidal anti-inflammatory drugs (NSAIDs), acetaminophen and opioids, are effective in treating acute pain [2].

NSAIDs contain two cyclooxygenase isoforms, namely, cyclooxygenase-1 (COX-1) and cyclooxygenase-2 (COX-2) [3]. COX-1 is constitutively expressed in tissues and promotes the synthesis of prostaglandin (PG). The gastric and renal side effects of NSAIDs may be due to their indirect influence on PGE2, which has a cytoprotective effect in the gastrointestinal system, as well as on PGE2 and PGI2, which regulate renal blood flow [4]. COX-2 is also located in certain healthy tissues, but this isoform is especially induced by inflammatory stimulus or mitogen in some tissues. The expression of COX-2 may be related to the synthesis of PG, which induces responses to pathological processes, such as pain, fever and inflammation [5]. Although nonselective NSAIDs are first-line analgesics, their additional inhibition of COX-1 increases the hazard of gastrointestinal toxicity and thus limits their administration [6].

Etoricoxib, as the COX-2 selective class of NSAIDs, can provide patients with effective painkillers who do not benefit adequately from first-line therapies [7]. As the second generation of the selective class of NSAIDs, in various cells and whole blood tests, etoricoxib is more than 100 times selective for COX-2 than for COX-1 and is obviously less active against COX-1 than other selective COX-2 inhibitors [8]. This drug is also effective in relieving pain during dental procedures, total abdominal hysterectomy, periodontal surgery and therapeutic knee arthroscopy [9-12]. This type of NSAID is a safe and effective drug that controls postoperative pain with minimal side effects.

The pre-emptive analgesic of large doses of etoricoxib (120 mg) has been demonstrated to reduce pain after tooth extraction surgery [13]. At present, few studies were conducted on the use of low-dose etoricoxib (60 mg) after the operation of impacted teeth, and the results cannot provide a basis for clinical practice.[1] Our clinical trial was performed to evaluate the efficacy of a low dose of etoricoxib (60 mg) on alleviating pain after third molar surgery.

**Working hypothesis**

The general working hypothesis is that the pain score of the test group is different from that of the control group during the 24-hour observation period. The special working hypothesis was defined as follows: the pain score of the test group within 24 hours is lower than that of the control group. The null hypothesis was that preventive use of low-dose etoricoxib has no effect on postoperative pain after extraction of impacted teeth.

**Aims of the study**

**Primary endpoint**

Pain score for 24 hours after tooth extraction

**Secondary endpoint**

The total dose of emergency analgesics used in each group

Number of people using emergency analgesics within 24 hours after tooth extraction

**Work flow of the study:**

Participants in the etoricoxib group took 60 mg of etoricoxib tablets orally half an hour before the operation, and then the impacted teeth were extracted. Participants in the placebo group took a placebo half an hour before the operation, and then the impacted tooth was extracted. After the operation, the patient will be given a survey score sheet and 3 ibuprofen capsules to guide the patient to fill in the score sheet correctly, and the electronic version of the score sheet will be sent back to the staff via WeChat 24 hours after the operation.

### Safety considerations:

All participants excluded surgical contraindications and those with COX-2 allergies. Emergency rescue medicines are prepared in the operating room under the guidance of senior doctors. After the participants take Etocoxib tablets or placebo, they will be accompanied by professional nurses and doctors throughout the process. In the event of an allergic reaction, timely symptomatic treatment will be given. All participants were observed for 1 hour after tooth extraction, no active bleeding or abnormalities, and allowed to go home.

**Follow-up:**

24 hours

**Study design:**

a randomized, parallel, double-blinded and placebo-controlled clinical trial

**Randomisation:**

The patients were divided into two groups (placebo and 60 mg of etoricoxib) by Excel software to achieve randomisation. Allocation concealment was maintained using a sealed opaque envelope. The research assistant prepared the study drugs for the [clinic](D:/360Downloads/Dict/8.9.3.0/resultui/html/index.html#/javascript:;) nurses according to the randomisation list. Clinic nurses, as non-treatment group members, gave the study drugs sealed in a similar package to the patients 30 minutes before the surgery.

**Inclusion criteria:**

Healthy patients scheduled to undergo surgical removal of an impacted horizontal mandibular third molar (Winter classification) were eligible for participation. Patients were included if they were older than 18 years old, had horizontal impacted teeth, did not take analgesics or anti-inflammatory drugs a week prior to the study.

**Exclusion criteria:**

Patients were excluded if they took NSAIDs and COX-2 inhibitors; were pregnant or nursing; had other serious diseases, such as liver, kidney and cardiovascular diseases; had ulcers or bleeding in the digestive tract; had inability to express subjective discomfort symptoms; and suffered from dental caries or apical periodontitis with the adjacent teeth.

**Termination of study:**

Individual termination of study, participant does not want to participate anymore.

**Statistical analysis**

Data were analysed with SPSS software (SPSS, Inc., USA). Independent t-test and Chi-square were used to determine significant difference between the two groups. The parametric outcomes were expressed as mean ± standard deviation (SD). The survival curves were estimated by the Kaplan–Meier method, and log-rank test was applied to compare differences between curves. *p* value less than 0.05 was considered statistically significant.

**Ethics**

The study was conducted according to the guidelines of the Declaration of Helsinki, and approved by the Ethics Committee of School & Hospital of Stomatology, Wuhan University (approval number 2019-B11). All participants informed of the benefits and risks of this trial in writing, and all participants signed an informed consent form

**References**

1. Malmstrom K, Sapre A, Couglin H, Agrawal NG, Mazenko RS, Fricke JR Jr. Etoricoxib in Acute Pain Associated with Dental Surgery: A Randomized, Double-Blind, Placebo- and Active Comparator–Controlled Dose-Ranging Study. Clin Ther. 2004;26:667-679.

2.Mehrabi M, Allen JM, Roser SM. Therapeutic agents in perioperative third molar surgical procedures. Oral Maxillofac Surg Clin North Am. 2007;19:69-84.

3.Seibert K, Zhang Y, Leahy K, Hauser S, Masferrer J, Isakson P. Distribution of COX-1 and COX-2 in normal and inflamed tissues. Adv Exp Med Biol. 1997;400A:167-170.

4. Süleyman H, Demircan B, Karagöz Y. Anti-inflammatory and side effects of cyclooxygenase inhibitors. Pharmacol Rep. 2007;59:247-258.

5. Meade EA, Smith WL, DeWitt DL. Differential inhibition of prostaglandin endoperoxide synthase (cyclooxygenase) isozymes by aspirin and other non-steroidal anti-inflammatory drugs. J Biol Chem. 1993;268:6610-6614

6. Wolfe MM, Lichtenstein DR, Singh G. Gastrointestinal toxicity of nonsteroidal antiinflammatory drugs. N Engl J Med. 1999; 340:1888-1899.

7. Brown JD, Daniels SE, Bandy DP, Ko AT, Gammaitoni A, Mehta A, Boice JA, Losada MC, Peloso PM. Evaluation of multiday analgesia with etoricoxib in a double-blind, randomized controlled trial using the postoperative third-molar extraction dental pain model. Clin J Pain. 2013;29:492-498.

8. Riendeau D, Percival MD, Brideau C, Charleson S, Dubé D, Ethier D, Falgueyret JP, Friesen RW, Gordon R, Greig G, Guay J, Mancini J, Ouellet M, Wong E, Xu L, Boyce S, Visco D, Girard Y, Prasit P, Zamboni R, Rodger IW, Gresser M, Ford-Hutchinson AW, Young RN, Chan CC. Etoricoxib (MK-0663): preclinical profile and comparison with otheragents that selectively inhibit cyclooxygenase-2. J Pharmacol Exp Ther. 2001;296:558-566.

9. Daniels SE, Bandy DP, Christensen SE, Boice J, Losada MC, Liu H, Mehta A, Peloso PM. Evaluation of the dose range of etoricoxib in an acute pain setting using the postoperative dental pain model. Clin J Pain. 2011;27:1-8

10. Viscusi ER, Frenkl TL, Hartrick CT, Rawal N, Kehlet H, Papanicolaou D, Gammaitoni A, Ko AT, Morgan LM, Mehta A, Curtis SP, Peloso PM. Perioperative use of etoricoxib reduces pain and opioid side-effects aftertotal abdominal hysterectomy: a double-blind, randomized, placebo-controlled phase III study. Curr Med Res Opin. 2011;28:1323-1335

11. Steffens JP, Santos FA, Sartori R, Pilatti GL. Preemptive dexamethasone and etoricoxib for pain and discomfort prevention after periodontal surgery: a double-masked, crossover, controlled clinical trial. J Periodontol. 2010;81:1153-1160

12. Lierz P, Losch H, Felleiter P. Evaluation of a single preoperative dose of etoricoxib for postoperativepain relief in therapeutic knee arthroscopy: a randomized trial. Acta Orthop. 2012;83:642-647.

13. Costa FW, Soares EC, Esses DF, Silva PG, Bezerra TP, Scarparo HC, Ribeiro TR, Fonteles CS. A split-mouth, randomized, triple-blind, placebo-controlled study to analyze the pre-emptive effect of etoricoxib 120 mg on inflammatory events following removal of unerupted mandibular third molars. Int J Oral Maxillofac Surg. 2015;44:1166-1174.

**Score sheet**

**Name:**

**Gender:**

**Age:**

**Weight (Kg):**

**Height (cm):**

**Visual Analogue Scale:**

1. **10**

**Pain score 2 hours after surgery:**

**Pain score 4 hours after surgery:**

**Pain score 6 hours after surgery:**

**Pain score 8 hours after surgery:**

**Pain score 12 hours after surgery:**

**Pain score 24 hours after surgery:**

**Total analgesic consumption for postoperative 24 hours (tablets):**

**Number of patients who consumed the first rescue analgesic medication during the period of evaluation (24 h):**
